# Delayed Progressive Mass Effect After Secured Ruptured Middle Cerebral Artery Aneurysm: Risk Factors and Outcomes

**DOI:** 10.3389/fsurg.2022.852576

**Published:** 2022-05-02

**Authors:** Ying-Ching Li, Ching-Chang Chen, Chun-Ting Chen, Po-Hsun Tu, Mun-Chun Yeap, Yi-Ming Wu, Zhuo-Hao Liu, Ting-Wei Chang, Ya-Jui Lin, Tai-Wei Erich Wu, Po-Chuan Hsieh

**Affiliations:** ^1^ Department of Neurosurgery, Chang Gung Memorial Hospital, Linkou, Chang Gung University and Medical College, Taoyuan, Taiwan; ^2^ Department of Neurosurgery, New Taipei Municipal Tucheng Hospital (Built and Operated by Chang Gung Medical Foundation), Taipei, Taiwan; ^3^ Department of Radiology, Chang Gung Memorial Hospital, Linkou, Chang Gung University and Medical College, Taoyuan, Taiwan

**Keywords:** brain oedema, middle cerebral artery, intracranial aneurysm, aneurysm clip, aneurysm embolization

## Abstract

**Objective:**

Delayed progressive mass effect (DPME) after securing an aneurysm is uncommon following microsurgical or endovascular repair and leads to a poor clinical outcome. Patients with ruptured middle cerebral artery (MCA) aneurysms have a high risk of postoperative oedema and mass effect, which may require decompressive treatment. Because few studies have discussed the risk and predictive factors, we focused on ruptured MCA aneurysms and evaluated the outcomes of these patients and the necessity of salvage surgery when DPME presented.

**Methods:**

Data on 891 patients with aneurysmal subarachnoid haemorrhage (aSAH) treated between January 2011 and February 2020 were extracted from the medical database of a tertiary referral centre. A total of 113 patients with aSAH resulting from at least one MCA aneurysm were identified. After excluding patients with several clinical confounders, we enrolled 80 patients with surgically treated aSAH. We examined the characteristics of aneurysms and hematomas, perioperative contrast pooling patterns, presence of distal hematomas, perisylvian low density, occlusive treatment modality, management strategies, the need for salvage surgical decompression, and postoperative 90-day outcomes to identify possible risk factors.

**Results:**

DPME was observed in 27 of the 80 patients (33.7%). The DPME and non-DPME group differed significantly in some respects. The DPME group had a higher risk of salvage surgery (*p* < 0.001) and poorer outcomes (mRS at day 90; *p* = 0.0018). The univariate analysis indicated that the presence of hematoma, CTA spot signs, perisylvian low density, and distal hematoma were independent risk factors for DPME. We also noted that DPME remained an independent predictor of a poorer 90-day functional outcome (mRS ≤ 2).

**Conclusion:**

DPME can lead to salvage decompression surgery and directly relates to poor outcomes for patients with a ruptured MCA aneurysm. Distal hematoma, perisylvian low density, and CTA spot signs on preoperative images can predict DPME.

## Introduction

Patients who develop the uncommon condition of delayed progressive mass effect (DPME) after an aneurysm is secured generally have poor outcomes (1). Ruptured middle cerebral artery (MCA) aneurysms with a large intraparenchymal or perisylvian hematoma lead to grave prognoses, with mortality rates exceeding 80% in patients who undergo nonoperative management (2–4). Urgent hematoma evacuation and aneurysm clipping have been the primary neurosurgical treatments, and long-term functional neurological outcomes are directly related to known risk factors, such as initial Hunt and Hess (H–H) grade, hematoma size, and hematoma location (2, 5–8). DPME caused by a ruptured aneurysm usually complicates patients’ recovery and may require additional neurosurgical decompression procedures, especially for ruptured MCA aneurysms (9–11). Although articles have indicated that DPME can cause catastrophic outcomes (11–14), few studies have examined the risk factors for DPME.

To our knowledge, this is the first and largest study to examine the development of DPME in patients after securing a ruptured MCA aneurysm. This study focused on patients with ruptured MCA aneurysms and evaluated the outcomes of these patients and the risk of salvage surgery after the development of DPME. We also identified the risk factors for DPME with preoperative computed tomography (CT); we report our experience managing DPME after securing ruptured MCA aneurysms to educate clinicians on the need to conduct secondary decompression procedures before treating ruptured MCA aneurysms.

## Methods

### Patients and Data Collection

Clinical data on patients surgically treated for aneurysmal subarachnoid haemorrhage (aSAH) between January 2011 and February 2020 were extracted from the medical database of a tertiary referral centre. We identified 113 patients with aSAH resulting from at least one MCA aneurysm.

We excluded patients who lacked early imaging evidence, discontinued treatment because of family matters, were bleeding from an unknown origin because of multiple aneurysms, and had a flow-related aneurysm caused by another vascular anomaly, such as arteriovenous malformation. Eighty patients with surgically treated ruptured MCA aneurysms were included for review.

We compared clinical severity, morphological characteristics of aneurysms and hematomas, perioperative contrast pooling patterns, presence of distal hematoma, perisylvian hypodensity, occlusive treatment modality, management strategies, including the need for salvage surgical decompression for an intracranial mass effect, and postoperative 90-day outcomes to identify possible risk factors for DPME.

### Clinical Management

All aneurysms were secured microsurgically or endovascularly within 48 h after presentation. Patients with preoperative clinical or radiographic signs of uncal herniation and patients with large hematomas in whom herniation was deemed imminent underwent emergent hematoma evacuation and aneurysm clipping. If they did not receive immediate surgery, patients with clinical or radiographic hydrocephalus or H–H grades III-V underwent external ventricular drain placement for cerebrospinal fluid (CSF) diversion and intracranial pressure (ICP) monitoring until definitive occlusive treatment for ruptured aneurysm was performed. Whether an additional craniectomy or lobectomy was necessary was determined intraoperatively on the basis of the size of the hematoma, the need for surgical exposure, and the degree of cerebral oedema. All patients underwent postoperative CT angiography (CTA) 1–2 weeks after initial treatment, and these follow-up images were examined for possible vasospasm. All patients were cared for in a dedicated neurovascular intensive care unit according to a standardised aSAH protocol (15). Informed consent was obtained from all patients after informing them in detail about the risks, benefits, and alternatives of the procedures with multidisciplinary decision-making. The study was approved by the Institutional Review Board (IRB no. 201800342B0).

### Term Definition

**Figure 1:**
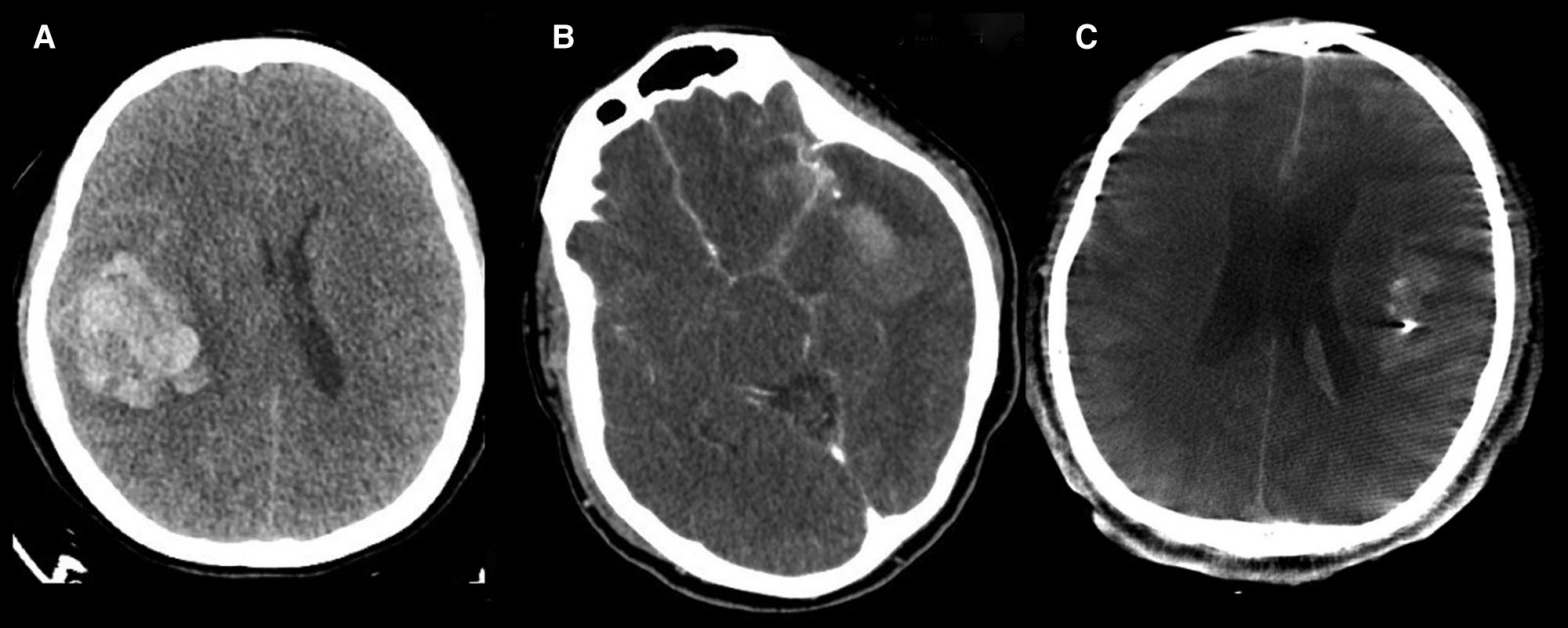
Image parameters documented on the initial brain CTA. (**A**) Distal hematoma. (**B**) Perisylvian low density. (**C**) CTA spot signs.

We studied the possible deterioration of a regional mass effect, whether haemorrhagic or ischemic; such an effect is rarely observed in patients after initial treatment for ruptured MCA aneurysms. DPME of a ruptured aneurysm is defined as the regional progression between the initial diagnostic CT and postoperative follow-up CTA. Potential predicted image parameters were documented on the basis of the image of the initial brain CT (**Figure 1**). The parameters comprised distal hematoma (intrasylvian hematoma at or cranially above the level of the third ventricle (**Figure 1A**)), perisylvian low density (>5 mm Low density area near the Sylvian fissure, detected through CT (**Figure 1B**)), and CTA spot signs (spot-like contrast extravasation noted on CTA (**Figure 1C**)).

### Statistical Analysis

Descriptive statistics were calculated for clinical and radiographic factors using the mean as a measure of central tendency. A univariate analysis and multivariate analysis of clinical characteristics and outcomes were conducted. We included some common outcome-related factors and some specific factors from our theory in our univariate analysis. Multivariate analysis was done by unconditional logistic regression: Association between factors and poor-functional outcome. All covariates with *p* value <0.2 in the univariate analysis, Y (poor-functional outcome) = PMERA + Age + pre-op Fisher + CTA spot sign + Perisylvian Low Density + Distal hematoma as reference). Contingency statistical analysis on categorical variables was performed with Fisher’s exact test. All statistical tests were two-sided, and *p* < .05 was prospectively determined to establish statistical significance. All analyses were performed using SPSS Statistics version 21.

## Results

### Patient Demographics and Radiographic Characteristics

The clinical data of 80 patients with aSAH caused by a ruptured MCA aneurysm were retrospectively analysed, and the detailed demographic and clinical characteristics are summarised in **Table 1**. The participants’ mean age was 54.2 years, and 31.2% were men. All patients were deemed functionally independent according to a medical record before presentation. The mean aneurysm size was 136.7 mm^3^, with a dome of 5.7 mm and neck of 2.9 mm, and all aneurysms were located at or proximal to the M1–2 bifurcation. A total of 26 (32.5%) patients had an aneurysm on the left, 28 (35%) patients had an H–H grade of IV or V at admission, and 46 (57.6%) patients had a Fisher grade of III or IV. A total of 46 (57.6%) patients presented with subarachnoid haemorrhage or intracranial haemorrhage. The mean hematoma volume was 13.6 mL, and the hematomas were entirely located on the ipsilateral side of the ruptured aneurysm. The mean of the maximum hematoma diameters was 24.5 mm. Preoperative images detected CTA spot signs in 25 (31.3%) patients, distal hematoma in 33 (41.2%) patients, and perisylvian low density in 33 (41.2%) patients. All patients were treated within 48 h. Microsurgical clipping was performed in 58 (72.5%) patients, and endovascular coiling was performed in 22 (27.5%) patients. Postoperative CT scans indicated 27 (33.7%) patients developed DPME. Of the 14 patients requiring salvage decompression surgery, 13 had DPME. Up to 48.1% patients with DPME required salvage surgery.

**Table 1:** Patient demographics and radiographic characteristics.

Characteristic	No.
Total patients	80
Age, year (SD)	54.2 (11.6)
Male	25 (31.2%)
Preoperative fisher grade	
1	22 (27.5%)
2	12 (15%)
3	13 (16.3%)
4	33 (41.3%)
Preoperative hunt and hess grade	
1	4 (5%)
2	21 (26.3%)
3	27 (33.8%)
4	20 (25%)
5	8 (10%)
Aneurysm size, (Mean/SD), mm^3^	136.7 (230.8)
Aneurysm dome (Mean/SD), mm	5.7 (2.9)
Aneurysm neck (Mean/SD), mm	2.9 (0.9)
Aneurysm dome/neck ratio (Mean/SD)	2.0 (0.8)
Wide neck (dome/neck ratio <1)	6 (7.5%)
Location (Left)	26 (32.5%)
Hematoma at presence (Yes)	46 (57.6%)
Hematoma, volume (Mean/SD), mL	13.6 (20.4)
Max hematoma diameter (Mean/SD), mm	24.5 (24.5)
Pre-op CTA spot sign^a^	
Yes	25 (31.3%)
No	55 (68.7%)
Distal hematoma^b^ (Yes)	33 (41.2%)
Perisylvian low density^c^ (Yes)	33 (41.2%)
DPME^d^	27 (33.7%)
Primary treatment	
Coiling	22 (27.5%)
Clipping	58 (72.5%)
Salvage operation (Yes)	14 (17.5%)

*Chi-square test (χ^2^ test); Independent Sample t test.*

*^a^* *CTA Spot Signs: Spot-like contrast extravasation noted on CTA.*

*^b^* *Distal Hematoma: Intrasylvian hematoma at or cranially above the level of the third ventricle.*

*^c^* *Perisylvian Low Density: >5 mm Low density area near the Sylvian fissure, detected through CT.*

*^d^* *DPME: Delayed progressive mass effect.*

### DPME and Other Radiographic Characteristics

Characteristics of the patients who developed DPME from MCA aSAHs are summarised in **Table 2**. DPME was observed in 27 of 80 (33.7%) patients with aSAHs caused by ruptured MCA aneurysms. A representative case of DPME is presented in **Figure 1**. A total of 19 (70.4%) patients who received surgical clipping and 8 (29.6%) patients who received endovascular coiling developed DPME after their primary treatment. The listed radiographic characteristics, including the morphology of the aneurysm, type of therapy (clipping or coiling), and preoperative Fisher and H–H grades, did not differ significantly between the DPME and non-DPME groups. However, some preoperative image factors, including hematoma size, distal hematomas, and perisylvian low density, differed significantly between these two groups (**Table 3**).

**Table 2:** Clinical characteristics within non-DPME and DPME groups.

Characteristics	Ruptured MCA (*n* = 80)		*p*-value
	Non-DPME^a^ (*n* = 53)	DPME^a^ (*n* = 27)	
Gender (Male)	19 (70.4%)	8 (29.6%)	0.5780
Age, year (SD)	54.2 (12.2)	54.0 (10.4)	0.9187
GCS in ER			0.0623
3–8	12 (22.6%)	13 (48.1%)	
9–12	11 (20.7%)	3 (11.1%)	
13–15	30 (56.7%)	11 (40.8%)	
Preoperative fisher grade			0.2365
1–2	25 (47.2%)	9 (33.3%)	
3–4	28 (52.8%)	18 (66.7%)	
Preoperative hunt and hess grade			0.2062
1–3	37 (69.8%)	15 (55.6%)	
4–5	16 (30.2%)	12 (44.4%)	
Preoperative Hemiplegia	17 (32%%)	13 (48.1%)	0.1603
Location (Left)	20 (37.7%)	6 (22.2%)	0.1613
Aneurysm size, (Mean/SD), mm^3^	120.1 (207.4)	169.3(272.3)	0.2708
Aneurysm dome, (Mean/SD), mm	5.5 (3.0)	6.1 (2.6)	0.1846
Aneurysm neck, (Mean/SD), mm	2.9 (0.9)	2.9 (1.0)	0.4562
Aneurysm dome/neck ratio (SD)	1.9 (0.8)	2.2 (0.9)	0.1847
Wide neck (dome/neck ratio <1)	4 (66.7%)	2 (33.3%)	0.9999
Hematoma (Mean/SD), mL	8.8 (18.1)	23.0 (21.8)	**<0.001**
Max hematoma diameter (Mean/SD), mm)	16.9 (22.2)	39.3 (22.2)	**<0.001**
CTA spot sign^b^ (yes)	6 (11.3%)	19 (70.3%)	**<0.001**
Distal hematoma^c^	15 (28.3%)	18 (66.7%)	**<0.001**
Perisylvian low density^d^	14 (26.4%)	19 (70.3%)	**<0.001**
Primary treatment (clipping)	39 (73.6%)	19 (70.4%)	0.7608
Salvage operation	1 (1.9%)	13 (48.1%)	**<0.001**
mRS at day 90 (Functional outcome)			**0.0018**
0–2	36 (67.9%)	8 (29.6%)	
3–6	17 (32.1%)	19 (70.4%)	
mRS at day 90 (Mortality)			0.1091
0–5	52 (98.1%)	24 (88.9%)	
6	1 (1.9%)	3 (11.1%)	

*Chi-square test (χ^2^ test); Independent sample t test.*

*^a^* *DPME: Delayed progressive mass effect.*

*^b^* *CTA Spot Signs: Spot-like contrast extravasation noted on CTA.*

*^c^* *Distal Hematoma: Intrasylvian hematoma at or cranially above the level of the third ventricle.*

*^d^* *Perisylvian Low Density: >5 mm Low density area near the Sylvian fissure, detected through CT.*

*Bold values indicate statistical significance.*

**Table 3:** DPME predictors after aSAH caused by ruptured MCA aneurysm.

Clinical data & image signs	Ruptured MCA (*n* = 80)		OR (95% CI)	*p*-value
	Non-DPME^a^ (*n* = 53)	DPME^a^ (*n* = 27)		
Age, y (SD)	54.2 (12.2)	54.0 (10.4)	0.998 (0.958–1.039)	0.917
Preoperative Fisher grade 3.4 (vs. 1.2)	28 (52.8%)	18 (66.7%)	1.786 (0.68–4.687)	0.239
Preoperative H-H grade 4–5 (vs. 1–3)	16 (30.2%)	12 (44.4%)	1.982 (0.752–5.22)	0.166
Aneurysm size, (Mean/SD), mm^3^	120.1 (207.4)	169.3 (272.3)	1.001 (0.99–1.003)	0.381
Primary treatment (clipping)	39 (73.6%)	19 (70.4%)	0.853 (0.305–2.382)	0.761
Hematoma (Mean/SD), mL	8.8(18.1)	23.0(21.8)	1.11 (1.053–1.171)	**<0.001**
Max hematoma diameter (Mean/SD), mm)	16.9(22.2)	39.3(22.2)	1.042 (1.02–10.7)	**<0.001**
CTA spot sign^b^ (yes)	6 (11.3%)	19 (70.3%)	6.86 (2.746–9.459)	**<0.001**
Distal hematoma^c^	15 (28.3%)	18 (66.7%)	5.067 (1.866–13.755)	**<0.001**
Perisylvian low density^d^	14 (26.4%)	19 (70.3%)	6.616 (2.368–18.481)	**<0.001**

*Univariate analysis. (Fisher’s exact test).*

^a^ *DPME: Delayed progressive mass effect.*

*^b^* *CTA Spot Signs: Spot-like contrast extravasation noted on CTA.*

*^c^* *Distal Hematoma: Intrasylvian hematoma at or cranially above the level of the third ventricle.*

*^d^* *Perisylvian Low Density: > 5 mm Low density area near the Sylvian fissure, detected through CT.*

*Bold values indicate statistical significance.*

Risk factors predictive of DPME, according to clinical data and images, are presented in **Table 3**. DPME was not significantly associated with age, primary treatment, preoperative H–H grade, Fisher grade, or aneurysm size. Rather, it was more highly associated with hematoma size (OR 1.11; 95% CI 1.053–1.171; *p* < 0.001), max diameter of the hematoma (OR 1.042; 95% CI 1.02–10.7; *p* < 0.001), CTA spot signs (OR 6.86; 95% CI 2.746–9.459; *p* < 0.001), distal hematoma (OR 5.067; 95% CI 1.866–13.755; *p* < 0.001), and perisylvian low density (OR 6.616; 95% CI 2.368–18.481; *p *< 0.001).

### DPME and Functional Outcomes

The Modified Rankin Scale (mRS) scores at 3 months after the initial aSAH event in the DPME and non-DPME groups are summarised in **Table 2**. Favourable functional outcomes (mRS scores of 0–2) were observed in 36 (81.8%) patients without DPME. The non-DPME group had significantly superior functional outcomes (*p* = 0.0018) to the DPME group.

Univariate logistic regression analysis demonstrated that the DPME group exhibited poorer functional outcomes (OR 5.029; 95% CI 1.836–13.774; *p* = 0.002; **Table 4**) than the non-DPME group. Perisylvian low density (OR 3.733; 95% CI 1.462–9.535; *p *= 0.006), distal hematoma (OR 3.733; 95% CI 1.462–9.535; *p* = 0.006), CTA spot signs (OR 6.43; 95% CI 1.548–9.183; *p* = 0.001), Fisher grade 3–4 (vs. 1–2; OR 3.947; 95% CI 1.509–10.327; *p* = 0.005), H–H grade 4–5 (vs. 1–3; OR 3.6; 95% CI 1.368–9.475; *p* = 0.009), and age (OR 1.046; 95% CI 1.003–1.091; *p *= 0.035) were also significant predictors of poor outcomes (**Table 4**).

**Table 4:** Multivariable analysis of possible outcome predictors.

Independent Factors	Odd Ratio	95% CI	*p*-value*
DPME	5.405	1.512–19.327	**0** **.** **009**
Age, y (per 10 increase)	1.760	1.002–3.092	**0**.**049**
Preoperative Fisher grade 3.4 (vs. 1.2)	3.008	0.901–10.041	0.073
Preoperative H-H grade 4–5 (vs. 1–3)	1.674	0.504–5.557	0.4
Perisylvian Low Density	0.979	0.081–11.787	0.986
Distal hematoma	3.175	0.213–47.289	0.402

*Y (treatment-related infarct) = GCS in ER + preoperative H–H + Preoperative Fisher + Hematoma presence (≥3 mL vs. <3 mL) + spot sign in preoperative and postoperative CT + perisylvian low density + distal hematoma.*

*All covariates with p value of <0.2 in univariate analysis.

*Bold values indicate statistical significance.*

## Discussion

### Ruptured MCA Aneurysm and DPME

**Figure 2:**
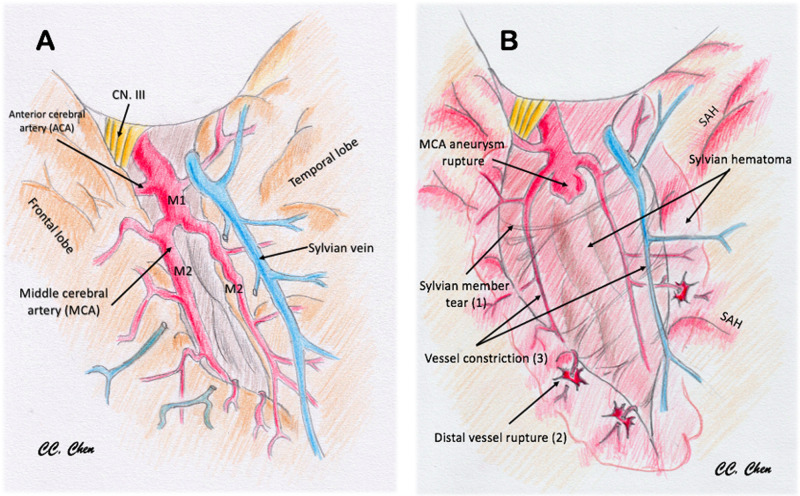
Illustration to demonstrate the pathomechanism of delayed progressive mass effect. (**A**) Normal vessels and sylvian fissure. (**B**) Pathological change after MCA aneurysm rupture with sylvian hematoma: (1) sylvian member and pia tear by the stretch of hematoma; (2) cortical artery was straightened and distal vessel rupture (spots sign or distal hematoma); (3) vessel diameter lessening, flow obstruction, and ischemia by hematoma compression (perisylvian low density).

Studies have reported that patients with aSAH with intraparenchymal hematomas usually present with higher H–H grades and have poorer functional neurological outcomes than patients with aSAH alone or with smaller hematomas (16). Nonoperative management for aSAH with large parenchymal hematomas and signs of uncal herniation is nearly universally fatal, whereas early hematoma evacuation and aneurysm clipping can improve short-term and long-term outcomes (2, 5, 6, 8). Studies have reported that patients with larger hematomas presented in poorer initial clinical condition and were more likely to require additional neurosurgical interventions involving decompressive craniectomy or hematoma evacuation (17). Smith et al. proposed a pathological process in which brain swelling and ischaemic infarction exacerbate each other in the limited intracranial space (11). Fisher and Ojemann described it as a “feed-forward” cycle of brain injury (9), and Saito et al. reported that patients with SAH with perisylvian hematoma can easily develop delayed postoperative brain swelling approximately 1 week after surgery (18). Perisylvian ICH was deemed to entail greater risk of developing postoperative brain oedema and infarction (**Figure 2**). A few case reports have identified multifocal extravasation sites from subpial MCA branches resulting in Sylvian hematoma formation. Sylvian hematomas may develop when bleeding occurs in the subpial space rather than in the subarachnoid space, and an expanding hematoma in this location can cause severe brain damage (1, 14). In this study, we identified possible risk factors that can alert neurosurgeons preoperatively and discussed the possible mechanisms resulting in haemorrhagic or ischaemic DPME after securing a ruptured MCA aneurysm.

### Possible Pathomechanism of DPME

ICH alone is a predictor of poor outcomes in association with ruptured MCA aneurysms (19–25). In such cases, the focal destruction of the functional brain area by an expanding hematoma volume results in possible permanent neurological deficits unrelated to the increased intracranial pressure that can be controlled through hematoma evacuation or other decompression procedures.

In our cohort, the incidence of postoperative brain swelling and infarction was 33% (27/80) in patients with ruptured MCA aneurysms. This incidence is relatively high compared with patients with other types of ruptured anterior circulation aneurysms. Several potential mechanisms have been proposed to explain the development of DPME. First, MCA aneurysms are usually located in a confined space within the Sylvian fissure that is adjacent to both the frontal and temporal lobes (26). Once an aneurysm ruptures, a dense focal hematoma usually forms near the lesion within the Sylvian fissure. When a thickened subarachnoid hematoma forms, small parasylvian cortical arteries are potentially dissected from the parasylvian brain parenchyma. Some studies have suggested that the sudden high pressure resulting from a ruptured aneurysm can damage the pial membrane and the parasylvian cortical arteries, which may result in a subpial extension of the subarachnoid hematoma. This phenomenon could explain the possible ischaemic effect of the blood supply to the parasylvian cortex being halted and the haemorrhagic effect due to punctuate bleeding from the disrupted end of the small cortical arteries. This phenomenon may also result in increased ischaemic or infarct core growth or delayed hematoma formation, as indicated by Yusuke et al. (26). However, we believe that because of the destruction of the microcirculation of the pia mater, this effect should be observed within a limited region of the parasylvian cortex without causing more severe primary vessel territory infarction. Furthermore, van Asch et al. (27) reported that the expansion of the ICH resulting from a ruptured aneurysm without signs of aneurysmal rebleeding is not rare within 48 h after onset, and they speculated that the hematoma expansion was caused by blood from damaged vessels surrounding the hematoma, which is consistent with our observations. These mechanisms were summarized and illustrated in **Figure 2**.

Second, the development of delayed cerebral ischaemia (DCI), the most critical complication of aSAH, has a severe impact on the outcome of patients; DCI has been widely studied. It accounts for almost 30% of new neurological deficits after an initial haemorrhage (10, 28). Yet, to date, the development of DCI cannot be predicted reliably for individual patients, and some early deterioration of the intracranial mass effect cannot be solely explained through vasospasm.

Third, peri-hematoma cerebral oedema after ICH is secondary to neuroinflammation and oxidative stress from reactions triggered by red blood cell lysis, thrombin production, complement activation, and neuroinflammation, which damage the blood–brain barrier (29–31). This delayed swelling is another possible mechanism of DPME.

Finally, we cannot exclude the possibility that these delayed effects on the brain could be secondary to the requirement for temporary occlusion of the parent artery during treatment of the ruptured aneurysm—the temporary clip used microsurgically or the balloon-assisted technique used endovascularly may be implicated. Therefore, concluding that a single mechanism is responsible for the condition of each patient with DPME is difficult.

### Practical Radiographic Predictors of DPME

**Figure 3:**
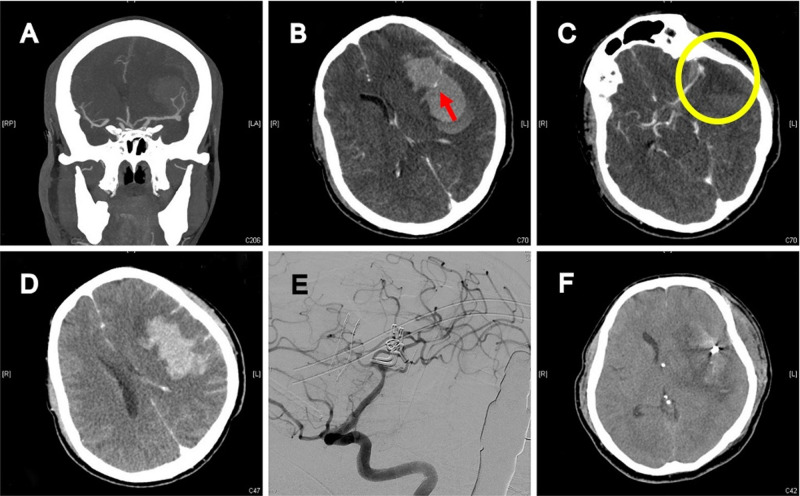
41-year-old female with high-grade aSAH caused by ruptured left MCA aneurysm. (**A**) Preoperative brain CTA depicted severe aSAH, ICH, and two MCA aneurysms. (**B**) Intra-hematoma spot sign (red arrow). (**C**) Perisylvian low density (yellow circle). (**D**) Distal hematoma. (**E**) 5 days after craniotomy to remove hematoma and clip two aneurysms; post-operative angiography revealed complete obliteration of aneurysms, well preservation of all MCA branches, and no vasospasm. (**F**) Delayed progressive mass effect with midline shift.

**Figure 4:**
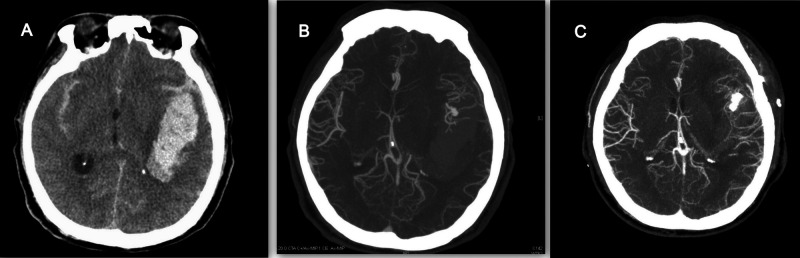
55-year-old female with ruptured left MCA aneurysm. (**A**) Large left temporal hematoma, but no perisylvian low density, no spot sign, no distal hematoma. (**B**) CTA revealed M1 bifurcation aneurysm. (**C**) After successful clipping, there were no delayed progressive mass effect 7 days after operation.

**Figures 3 and 4** demonstrated the contrast results of clipping MCA aneurysm with different preoperative CTA findings. Univariate analysis revealed significant differences in CTA spot signs, distal hematoma, and perisylvian low density between the DPME and non-DPME groups (**Table 3**). We also demonstrated that hematoma size and maximum hematoma diameter are significant risk factors of DPME. This finding is similar to ﻿﻿that of Hilditch et al. (1), who stated that sylvian hematomas may develop secondary to bleeding in the subpial space rather than in the subarachnoid space and an expanding hematoma in the subpial space can cause severe brain damage. When an aneurysm adherent to the pia mater bleeds, the blood may spread below the pia, and the expanding hematoma may rupture the thin arteries that run in the pial space. Suzuki et al. (32) indicated that patients in three studies had either MCA aneurysms or posterior communicating artery aneurysms and a ruptured aneurysm close to the Sylvian fissure may result in blood flowing into the subpial space and triggering events leading to hematoma formation. Therefore, in light of the literature and our findings, we propose that neurosurgeons should note these potential preoperative predictors and prepare for the possible subsequent deterioration of intracranial mass effects and the need for early decompression and extensive hematoma evacuation.

### DPME and Functional Outcomes

The outcome of a patient with a ruptured aneurysm depends heavily on the initial GCS score; however, other factors, such as age, vasospasm, collateral vessel perfusion, and hematoma volume, may alter the outcome (33). In this study, the outcomes between the DPME and non-DPME group differed significantly (*p* = 0.0018; **Table 2**); moreover, in our cohort, 14 (17.5%) patients required salvage surgery, but the rate of those requiring salvage surgery was significantly higher (48.1%) in the DPME group (*p* < 0.001; **Table 2**). Only one patient in the non-DPME group required salvage surgery because he received the surgery as his level of consciousness diminished. We determined other possible risk factors, including age, initial H–H and Fisher grades, volume of hematoma, CTA spot signs, perisylvian low density, distal hematoma, and DPME, through a univariate logistic regression analysis (**Table 5**). Neither the morphology of the aneurysm or a wide-neck aneurysm appears to greatly affect patients’ outcomes.

**Table 5:** Outcome predictors after aSAH caused by ruptured MCA aneurysm.

Variable factors	Functional outcome (*n* = 80)		OR (95% CI)	*p*-value^a^
	favorable (*n* = 44)	Poor (*n* = 36)		
Age, y (SD)	51.6 (10.3)	57.3 (12.4)	1.046 (1.003–1.091)	**0.035**
Preoperative Fisher grade 3.4 (vs. 1.2)	19 (43.9%)	27 (75%)	3.947 (1.509–10.327)	**0.005**
Preoperative H-H grade 4–5 (vs. 1–3)	10 (22.7%)	18 (50%)	3.6 (1.368–9.475)	**0.009**
Aneurysm size, (Mean/SD), mm^3^	148.7 (227.6)	122.0 (237.0)	0.999 (0.997–1.002)	0.608
Aneurysm dome, (Mean/SD), mm	5.7 (2.7)	5.6 (3.1)	0.993 (0.851–1.159)	0.926
Aneurysm neck, (Mean/SD), mm	3.0 (0.9)	2.8 (0.9)	0.856 (0.521–1.407)	0.540
Wide neck (dome/neck ratio <1)	3 (6.8%)	3 (8.3%)	1.242 (0.235–6.565)	0.789
Primary treatment (clipping)	31 (70.5%)	27 (75%)	1.258 (0.465–3.4)	0.651
Hematoma (Mean/SD), mL	8.07 (10.4)	14.15 (10.26)	1.058 (1.012–1.106)	**0.014**
CTA spot sign^a^ (yes)	6 (13.6%)	19 (52.8%)	6.43 (1.548–9.183)	**<0.001**
Distal hematoma^b^	12 (27.2%)	21 (58.3%)	3.733 (1.462–9.535)	**0.006**
Perisylvian low density^c^	12 (27.2%)	21 (58.3%)	3.733 (1.462–9.535)	**0.006**
DPME^d^	8 (18.2%)	19 (52.8%)	5.029 (1.836–13.774)	**0.002**

*Univariate analysis. (Fisher’s exact test).*

*^a^* *CTA Spot Signs: Spot-like contrast extravasation noted on CTA.*

*^b^* *Distal Hematoma: Intrasylvian hematoma at or cranially above the level of the third ventricle.*

*^c^* *Perisylvian Low Density: >5 mm Low density area near the Sylvian fissure, detected through CT.*

*^d^* *DPME: Delayed progressive mass effect.*

*Bold values indicate statistical significance.*

### Limitations of the Study

Selection bias and lack of randomisation are two limitations of this retrospective study. The role of vasospasm in the development of DPME is underestimated because our study mandated that a minority of patients who required intra-arterial therapy for vasospasm. Additionally, operator-dependent matters, such as the timing and method of temporary occlusion during the procedure, were still variable. However, in the study, the procedures were all performed by attending neurosurgeons and neurointerventionalists with at least 5-year experience of aneurysm treatment in the single center. Therefore, the general principles and protocols, including postoperative care, image follow up, nimodipine, fluid and blood pressure control for vasospasm, were consistent and coherent. The bias should be minimum. A well-designed prospective validation of independent cohorts is required to determine the exact timing of and indication for extra decompression procedures when DPME is anticipated. Otherwise, a multicenter investigation would be considered to establish more population and better confidence with normal distribution.

## Conclusion

DPME is directly related to poor outcomes in patients with a ruptured MCA aneurysm. Furthermore, these patients might have a higher incidence of secondary salvage decompression procedures. Examining preoperative images for prognostic characteristics, such as distal hematoma, perisylvian low density, and CTA spot signs, is recommended to predict DPME.

## Data Availability

The raw data supporting the conclusions of this article will be made available by the authors, without undue reservation.
